# Ru-specific promotion in electrodeposited Pt-based bimetallic catalysts for isopropanol electrooxidation

**DOI:** 10.1039/d6ra06258j

**Published:** 2026-09-07

**Authors:** Yuxuan Shi, Jingjing Huang, Haoyu Zhao, Gao Chen, Dongsheng Geng, Hongen Yu

**Affiliations:** a Jiangsu Key Laboratory of New Energy Devices & Interface Science, School of Chemistry and Materials Science, Nanjing University of Information Science & Technology (NUIST) Nanjing 210000 China yuhongen@nuist.edu.cn

## Abstract

Isopropanol/acetone is a promising electroactive liquid organic hydrogen carrier pair due to its high hydrogen storage capacity (3.4 wt%), low toxicity, and large-scale availability. The slow electrocatalytic oxidation of isopropanol is the bottleneck for the isopropanol/acetone pair's energy-storage applications. Platinum-based catalysts have shown distinguished catalytic activity in this reaction but suffer from catalyst poisoning and performance degradation. Second-metal doping, especially ruthenium, is considered a common strategy to enhance catalytic performance. To clarify whether the enhancement originates from a general bimetallic effect or a ruthenium-specific promotion, this study prepared platinum–ruthenium and platinum–rhodium catalysts with varying metal ratios *via* electrodeposition and evaluated them for isopropanol electrooxidation. Electrochemical analysis reveals that ruthenium doping is superior to rhodium doping, and that a 1 : 1 atomic ratio is optimal. Kinetic and thermodynamic investigations reveal that both alterations in the electronic structure and ruthenium-specific oxygen affinity contribute to the enhanced efficiency and stability of bimetallic catalysts in the oxidation of isopropanol, with the latter being more pronounced.

## Abstract 2

Ruthenium doping improves the catalytic performance of the Pt-based catalyst for the electrochemical oxidation of the promising electroactive liquid organic hydrogen carrier isopropanol, *via* both bimetallic synergy and oxygen affinity.

## Graphical abstract

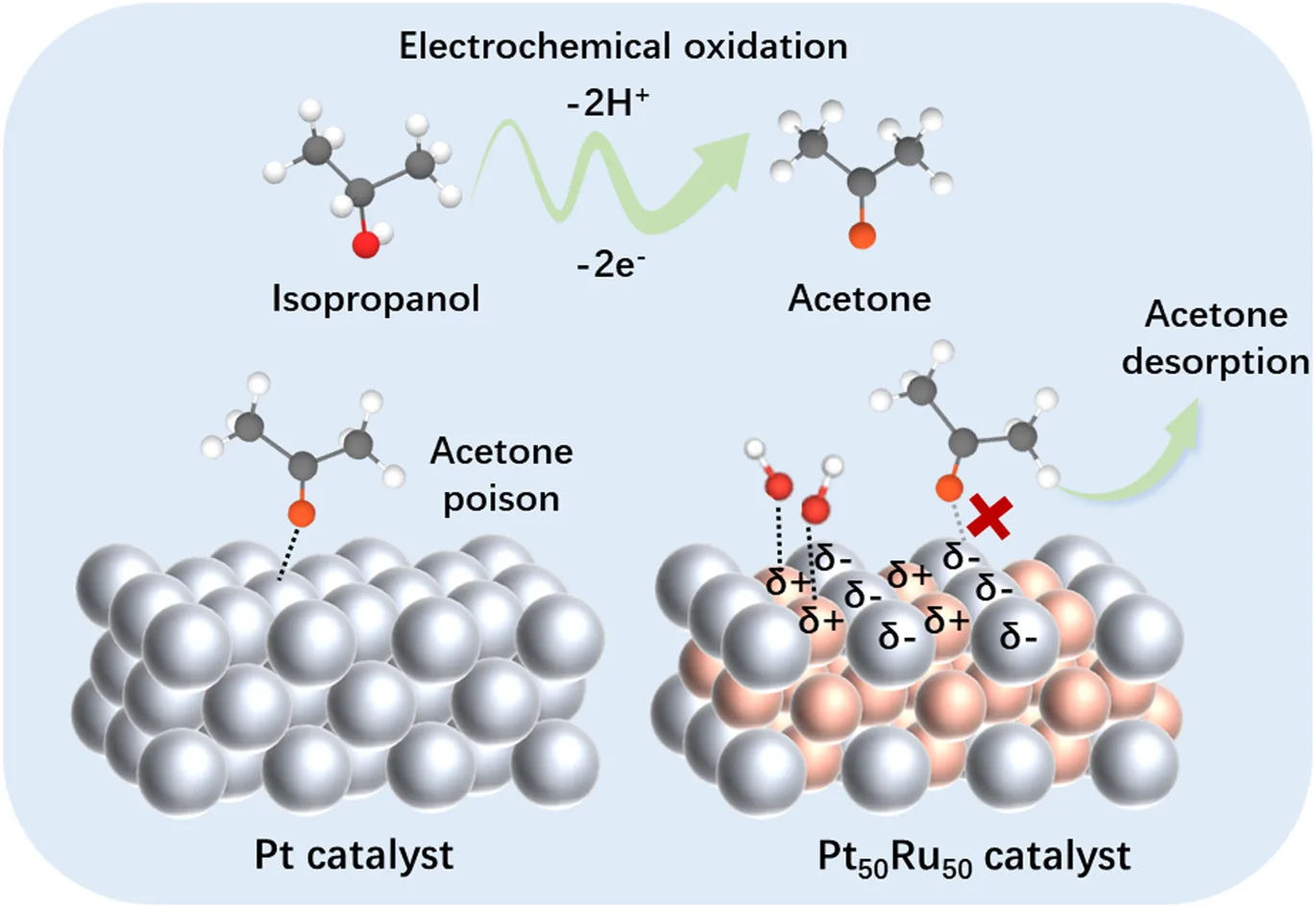

## 1. Introduction

With ever-increasing global energy demand and the pressing environmental challenges associated with fossil fuel consumption, the development of clean, sustainable energy alternatives has become imperative.^1^ Liquid organic hydrogen carrier (LOHC) technology has emerged as a promising solution that leverages existing infrastructure for the safe and efficient storage and transportation of hydrogen.^2–6^ However, conventional thermo-catalytic LOHC dehydrogenation is hampered by significant drawbacks, including high energy input and severe operating conditions.^7–10^ Electrocatalytic LOHC dehydrogenation presents a viable alternative, operating at relatively mild temperatures (<80–100 °C) and pressures (<0.1–0.5 MPa).^11–13^ Moreover, this approach enables the direct use of LOHCs as liquid fuels in fuel cells, thereby facilitating the reversible utilization of organic fuels. Compared with fully decomposed organic fuels, such as methanol and ethanol, electrocatalytic LOHC enables the reversible utilization of organic fuels *via* a pathway that circumvents direct CO_2_ formation.^14^

Among electrocatalytic LOHC systems, the isopropanol/acetone pair has attracted considerable attention.^15^ Isopropanol is soluble, relatively non-toxic, and can be produced from renewable biomass; the transformation between the pair is highly selective, with limited side reactions.^16^ When adapting the isopropanol/acetone pair for fuel cells and aqueous batteries, the electro-oxidation doesn't break the C–C bond, and the only dehydrogenation product is acetone, thereby avoiding severe catalyst poisoning by intermediate CO. Moreover, this system exhibits limited fuel permeation, laying the foundation for future integration into fuel cells and electrolyzers.^11,17^ In applications such as novel aqueous batteries, isopropanol-based secondary batteries achieve a charge capacity of 525 mA h g^−1^ at a 1C rate, with charge–discharge efficiency maintained at 95%.^18^ Nevertheless, the implementation of high-performance direct isopropanol fuel cells hinges on the development of efficient anode catalysts capable of overcoming the slow kinetics of isopropanol electro-oxidation (IOR).^19^

In acidic media (0.1 M HClO_4_ or 0.1 M H_2_SO_4_), platinum-based catalysts exhibit nearly 100% selectivity for the conversion of isopropanol to acetone when the potential is below 0.8 V_RHE_; when the potential rises above 0.8 V_RHE_, only trace amounts of CO_2_ are detected as a by-product.^20–23^ It suffers from a critical limitation: the adsorption of acetone molecules on Pt active sites leads to catalyst poisoning and performance degradation.^14^ Acetone's poisoning effect is not as severe as CO, but it still poses a challenge. To tackle this issue, the construction of Pt–M (M for a second transition metal) bimetallic catalysts has been widely adopted as an effective strategy to tailor surface adsorption properties. This approach leverages both electronic effects, in which the second metal M modulates the Pt d-band center to weaken acetone adsorption, and synergistic effects, in which M sites facilitate the adsorption of oxygenated species that promote the oxidative desorption of acetone. Some of the common oxyphilic elements have been investigated as M, *e.g.*, Ru,^24–26^ Rh,^27^ Bi, and Sb.^28^ For instance, Chelaghmia *et al.*^24^ demonstrated that Pt–Ru catalysts exhibit enhanced IOR activity, as the secondary metal adsorbs hydroxyl species at lower potentials than Pt, aiding the oxidative removal of adsorbed intermediates and regenerating active Pt sites. Similarly, Waidhas^29^ and Khanipour^30^ reported that Pt–Ru catalysts initiate isopropanol oxidation at an exceptionally low potential of 0.05 V_RHE_, underscoring their superior activity in the low-potential regime. Rh is another potential oxygen-affinitive doping element. Rh-based electrocatalysts have been widely used in methanol and ethanol electrooxidation,^27^ and ultrathin Rh nanosheets have been proven effective for isopropanol electrooxidation in KOH.^31^ These findings collectively highlight the efficacy of bimetallic synergism and surface engineering in advancing the electrocatalytic performance of isopropanol oxidation. In this study, Pt–Ru and Pt–Rh bimetallic catalysts with varying Pt/M molar ratios were synthesized and applied to the electrocatalytic oxidation of isopropanol under acidic conditions. Ru and Rh were selected as representative doping metals because they can modulate the electronic structure of Pt and interact with oxygenated surface species, while exhibiting different oxygen affinities. Ru is well-known for its oxyphilic nature and its ability to promote the formation of surface OH species at relatively low potentials; Rh, in contrast, provides a useful noble-metal reference to distinguish the Ru-specific promotional effect from a general bimetallic or alloy effect. It turns out that Ru doping exhibits superior catalytic performance to Rh doping, and the Pt_50_Ru_50_ catalyst achieves optimal performance, with a low onset potential of approximately 0.1 V_RHE_ and a peak current density of 11 mA cm^−2^. While the bimetallic effect of Pt–Rh promotes isopropanol electrooxidation to a certain extent, the lack of oxygen affinity on Rh hinders further enhancement of electrocatalytic performance.

## 2. Experimental section

### 2.1. Chemicals and materials

All the chemicals are of analytical grade and used without further purification. Nitric acid (HNO_3_, 69%), perchloric acid (HClO_4_, 70%), and the percentage here refers to concentration, potassium chloroplatinate (K_2_PtCl_6_, ≥98%), ruthenium(iii) chloride trihydrate (RuCl_3_·3H_2_O, ≥99.9%), rhodium(iii) chloride trihydrate (RhCl_3_·3H_2_O, ≥99.9%), hydrochloric acid (HCl, 37%), and isopropanol (IPA, 99.5%) were purchased from Sigma-Aldrich. Hydrophilic carbon fiber paper (CFP, TGP-H-090) was purchased from Suzhou Sinero New Energy Technology Co. Deionized (DI) water with a resistance of 18.25 MΩ cm^−1^ was used in all experiments.

### 2.2. Preparation of Pt_*x*_M_*y*_ (M = Ru, Rh) electrocatalysts

Pt_*x*_M_*y*_ (M = Ru, Rh) alloy catalysts were prepared *via* co-electrodeposition at a constant potential of −1 V_RHE_ for 30 min in a three-electrode system. A 1 × 2 cm^2^ hydrophilic CFP was used as the working electrode, with a platinum plate and an Ag/AgCl electrode as the counter and reference electrodes, respectively. CFP was ultrasonically pretreated sequentially in acetone and ethanol, then rinsed 3 times with DI water. The deposition electrolyte of Pt_*x*_Ru_*y*_ consisted of K_2_PtCl_6_ and RuCl_3_·3H_2_O with different Pt/Ru molar ratios (3 : 1, 2 : 2, and 1 : 3) in a 30 mL aqueous solution with a total metal ion concentration of 0.2 mM. The pH of the solution was adjusted to 2.0 using HCl. During electroreduction, N_2_ was continuously bubbled at a flow rate of 30 sccm. The obtained Pt_*x*_Ru_*y*_ on CFP was rinsed with DI water and then dried at 60 °C in a vacuum oven for 6 hours. The preparation of monometallic Pt and Ru catalysts follows the same procedure, except that a single-component solution of 0.2 mM K_2_PtCl_6_ or RuCl_3_·3H_2_O was used, respectively. By weighing the CFPs before and after electrodeposition, the loadings of Pt_*x*_Ru_*y*_, Pt, and Ru deposited onto them were maintained at 1.8 mg cm^−2^. To prepare the Pt_*x*_Rh_*y*_ and Rh catalysts, simply replace RuCl_3_·3H_2_O with RhCl_3_·3H_2_O; the other steps remain the same.

The synthesis of catalysts on the glassy carbon electrode was basically the same (geometric area: 0.196 cm^2^). The difference was that the glassy carbon electrode was used as the working electrode, and the pretreatment included polishing it with alumina powder and ultrasonic cleaning in ethanol and DI water. The concentration of metal ions in the electrolyte solution was reduced to 0.02 mM, and the deposition time was shortened from 30 min to 10 min, scaled proportionally to the surface areas of the glassy carbon and carbon paper electrodes.

### 2.3. Characterizations

The as-prepared catalysts were characterized through field emission scanning electron microscopy (SEM), transmission electron microscopy (TEM, JEOL-2100F), with elemental mapping using Oxford X-MaxN 80T IE250, X-ray diffraction (XRD, Smartlab, Rigaku), inductive-coupled plasma atomic emission spectroscopy (ICP-AES, Agilent 700), X-ray photoelectron spectrometry (XPS, Thermo Scientific, Nexsa G2). TEM specimens were prepared as follows: the as-prepared Pt_*x*_M_*y*_ (M = Ru, Rh) electrodes were ultrasonicated for 30 min in deionized water, and then 10 µL of the dispersion was dropped onto a copper mesh with an ultrathin carbon film, which was then dried in air.

### 2.4. Electrochemical measurements

The electrochemical tests were performed using a typical three-electrode system connected to the biologic electrochemical workstation. The glassy carbon electrode, Ag/AgCl electrode (filled with saturated aqueous KCl solution), and platinum plate were used as working, reference, and counter electrodes, respectively. During all measurements, N_2_ was continuously bubbled into the electrolyte to exclude air. All potentials measured were referenced against the reversible hydrogen electrode (RHE): *E* (V *vs.* RHE) = *E* (V *vs.* Ag/AgCl) + 0.0591 V × pH + 0.197 V. Note that the change in the electrolyte pH during the reaction is negligible. The current density was normalized to the geometric electrode area (0.196 cm^2^), and the potential was not *iR* compensated, unless otherwise specified. The electrolyte consists of a 0.1 M perchloric acid solution containing 1 M isopropanol. Before the test, the working electrode was electrochemically cleaned by cycling it 50 times between 0 and 1.5 V at 50 mV s^−1^. Prior to isopropanol oxidation, a pre-cleaning process was performed to remove any adsorbed carbon-containing material from the electrode surface, involving the sequential application of 1.5 V for 1 min and 0.0 V for 4 min.^32^ Cyclic voltammetry (CV) scans were conducted over a potential range of 0.0 to 1.0 V, where the faradaic current was observed, at scan rates of 10, 50, 100, 150, 200, 250, and 300 mV s^−1^. Linear sweep voltammetry (LSV) was performed at a scan rate of 5 mV s^−1^. Electrochemical impedance spectroscopy was measured over a frequency range of 0.01 Hz to 100 kHz at an applied potential of −0.2 V. It is worth noting that the potential of −0.2 V_RHE_ selected for the EIS here is intended to compare the relative strengths and weaknesses of the intrinsic charge transfer capabilities. The ratio of forward to reverse peak currents was calculated to assess whether the reaction was diffusion-controlled. CV scans were conducted over a potential range of 0.0 to 1.0 V at temperatures of 293 K, 303 K, 313 K, 323 K, and 333 K. The catalytic activity of electrocatalytically active isopropanol samples with different ratios was compared by calculating the activation energy. EIS measurements were performed at an AC amplitude of 5 mV over the frequency range of 10^−2^ Hz to 10^5^ Hz. Stability testing was conducted at 0.5 V to compare the stability of isopropanol electrocatalytic oxidation across different sample ratios.

## 3. Results and discussion

### 3.1. Characterization of the catalyst

The crystal structures of the electrodeposited Pt_*x*_M_*y*_, Pt, and M (M = Ru, Rh) were characterized by X-ray diffraction (XRD). The XRD pattern of Ru (Fig. 1a) shows five broad diffraction peaks in the 35° to 75° range, attributed to the hexagonal close-packed (hcp) structure. The diffraction peaks of Ru appear at 2*θ* values of 38.4° and 44.0°, corresponding to the (111) and (101) crystal planes of the (hcp) Ru (PDF # 97-005-2004), respectively. The Pt diffraction peaks (Fig. 1a) manifest at 2*θ* values of 39.8°, 46.2°, and 67.5°, corresponding to the (111), (200), and (220) crystal planes of Pt (PDF # 97-018-0880). The XRD pattern of Pt_50_Ru_50_ (Fig. 1a) indicates that Pt–Ru forms an alloy that predominantly adopts a Pt-based structure, with Ru doped into the Pt lattice. The lattice parameters a are 3.91 Å, 3.88 Å, and 3.86 Å (for cubic systems, *a* = *b* = *c*) for Pt, Pt_75_Ru_25_, and Pt_50_Ru_50_, respectively (Table S1). The lattice constant of the Pt_*x*_Ru_*y*_ samples decreases with increasing Ru doping, indicating that Ru doping induces lattice contraction due to the smaller atomic radius of Ru (1.34 Å) relative to Pt (1.39 Å).^33^ The diffraction peaks of Rh appear at 2*θ* values of 41.1°, 47.8°, and 69.9°, corresponding to the (111), (200), and (220) crystal planes of the (fcc) Rh (PDF # 97-065-0218), respectively (Fig. 1b). The calculated lattice parameters a are 3.91 Å, 3.88 Å, and 3.87 Å (for cubic systems, *a* = *b* = *c*) for Pt, Pt_75_Rh_25_, and Pt_50_Rh_50_, respectively (Table S2). Similar to Pt_*x*_Ru_*y*_, the alloy consists primarily of a Pt lattice with Rh dopants.^34^

**Fig. 1:**
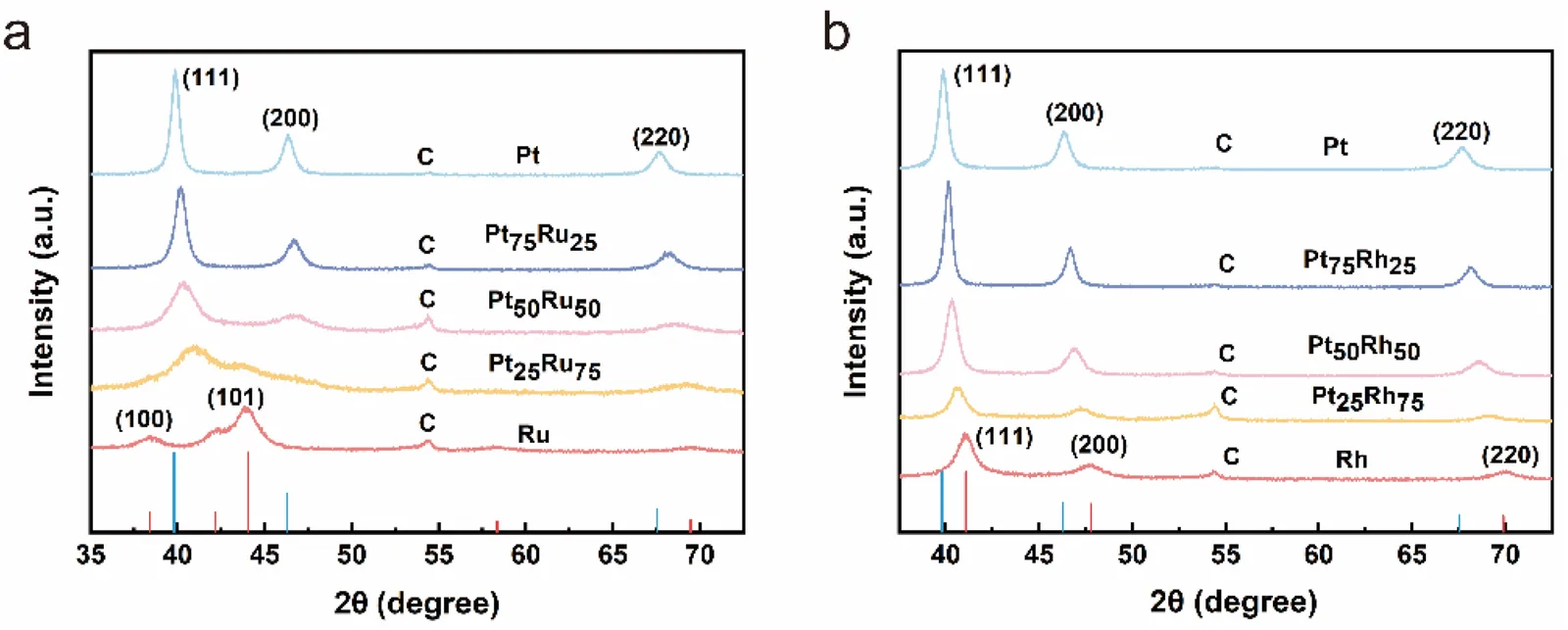
(a) XRD patterns of Ru, Pt, and Pt_*x*_Ru_*y*_. (b) XRD patterns of Rh, Pt, and Pt_*x*_Rh_*y*_.

Quantitative analysis of Pt and M (M = Ru, Rh) elements in the catalyst was performed using the inductive-coupled plasma atomic emission spectroscopy (ICP-AES). ICP-AES results indicate discrepancies between the measured catalyst composition and the nominal feed ratio (Table S3), likely due to incomplete dissolution of alloy ruthenium using acid. However, the elemental content estimated by electron microscopy is rather close to the feeding ratio (Table S4), confirming the above assumptions. Similarly, the measured Pt/Rh atomic ratios for the Pt_75_Rh_25_ and Pt_50_Rh_50_ samples differ from the feed ratios (Table S4), indicating that Rh may also suffer from the incomplete dissolution problem. To simplify the expressions, the as-synthesized Pt_*x*_M_*y*_ (M = Ru, Rh) catalysts are referred to by their feeding ratio in the following text.

The particle sizes and morphology of Pt_*x*_M_*y*_ (M = Ru, Rh) alloys were characterized by scanning electron microscopy (SEM). Pure deposited Pt exhibits distinct nanosheet structures, while pure deposited ruthenium appears to have granular structures. Pure deposited Rh, like pure Pt, exhibits a flower-like structure formed by the stacking of layers (Fig. S1). In contrast, Pt_75_Ru_25_, Pt_50_Ru_50_, and Pt_25_Ru_75_ all display granular structures (Fig. 2a–c), indicating that Ru doping significantly alters the alloy's growth mechanism. Among these, the pure Ru sample possesses the smallest particle size, with an average diameter of approximately 60 nm, suggesting higher dispersion (Fig. S2). The particle size of the Pt–Ru alloy samples is significantly larger than pure Ru. The Pt_75_Ru_25_ sample has an average diameter of 110 nm, and the Pt_50_Ru_50_ sample has an average diameter of around 140 nm. While the Pt_25_Ru_75_ sample is about 130 nm. Unlike Ru-doped samples, the morphology of the Pt_*x*_Rh_*y*_ alloy lies between that of pure Pt and pure Rh, both of which consist of layered stacks (Fig. 2d, e and S1).

**Fig. 2:**
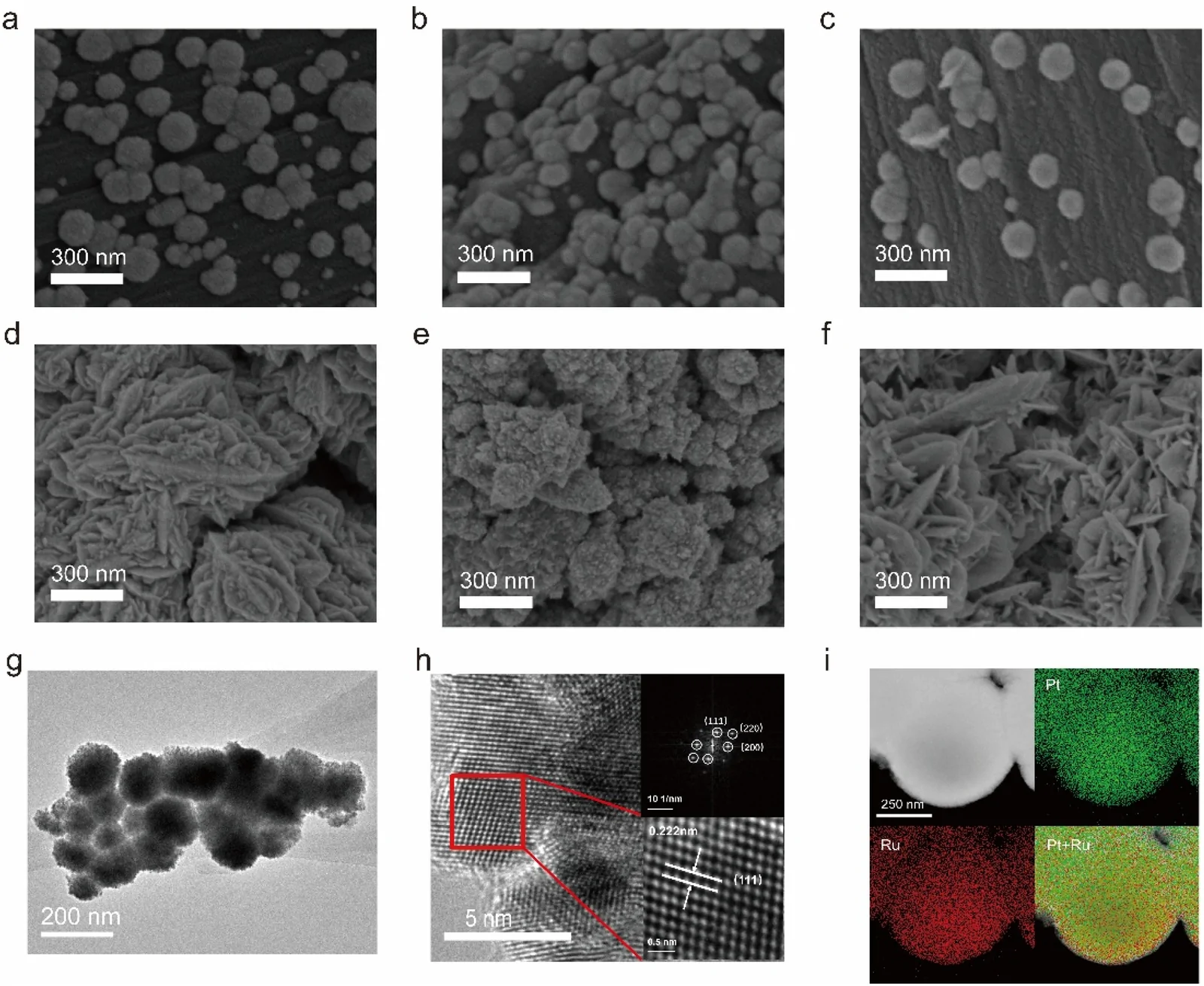
SEM images of Pt_*x*_M_*y*_: (a) Pt_75_Ru_25_, (b) Pt_50_Ru_50_, (c) Pt_25_Ru_75_, (d) Pt_75_Rh_25_, (e) Pt_50_Rh_50_, (f) Pt_25_Rh_75_. TEM images of Pt_50_Ru_50_ and EDS mapping: (g)–(i).

High-resolution transmission electron microscopy (HRTEM) (Fig. 2g and h) reveals that the Pt_50_Ru_50_ sample exhibits a compact, clustered distribution. Transmission electron microscopy-energy dispersive spectroscopy (TEM-EDS) elemental mapping (Fig. 2i) reveals a uniform distribution of Pt and Ru within the alloy. The specific element content is shown in Table S4, with a Pt/Ru atomic ratio of approximately 1.36 : 1, confirming our speculation of incomplete Ru dissolution and explaining the relatively low Ru content in the ICP-AES results. Under higher magnification (Fig. 2h), lattice spacings of 0.222 nm, 0.135 nm, and 0.195 nm for the (111), (220), and (200) crystal planes of Pt (PDF # 97-018-0880) can be distinguished, respectively.

### 3.2. Electrooxidation activity for isopropanol on Pt_*x*_M_*y*_ catalysts

In the electrochemical catalysis experiments, glassy carbon electrodes were employed to evaluate Pt_*x*_M_*y*_ (M = Ru, Rh) catalysts at varying ratios towards isopropanol electro-oxidation in a 0.1 M perchloric acid electrolyte. Pt and Pt_*x*_M_*y*_ (M = Ru, Rh) all exhibit characteristic isopropanol oxidation peaks during both forward and reverse CV scans (Fig. 3a and d). The initial potential of the Pt sample is 0.3 V_RHE_, with the peak current potential for isopropanol electrooxidation occurring at 0.6 V_RHE_. The oxidation peak during the forward scan corresponds to the oxidation of isopropanol to produce acetone, whilst the peak during the reverse scan corresponds to the same reaction catalyzed by regenerated Pt active sites after negative-potential cleansing of acetone poisoning.^35^ As Ru and Rh alone lack the ability to catalyze isopropanol oxidation, no isopropanol oxidation peak is observed on the Ru and Rh samples. All the Pt_*x*_M_*y*_ (M = Ru, Rh, *x* + *y* = 100) alloy samples exhibit lower onset potentials. The onset potential for Pt_*x*_Ru_*y*_ is 0.1 V_RHE_, whereas that for Pt_*x*_Rh_*y*_ is 0.05 V_RHE_. Among them, the Pt_50_Ru_50_ sample exhibits the highest peak current density, reaching 11 mA cm^−2^, whereas the Pt_50_Rh_50_ sample is significantly lower at 0.7 mA cm^−2^. The forward scan of isopropanol oxidation on the Pt_*x*_Ru_*y*_ alloy is divided into two regions (peaks at 0.2 V_RHE_ and 0.6 V_RHE_), corresponding to the electrooxidation of isopropanol at Pt–Ru bimetallic sites and Pt–Pt sites, respectively.^24^ As shown in LSV in Fig. S3a, Pt_50_Ru_50_ achieves a current density of 2.2 mA cm^−2^ at 0.2 V_RHE_, markedly higher than that of Pt, Pt_75_Ru_25_, and Pt_25_Ru_75_. Pt_50_Ru_50_ achieves the optimal Pt–Ru synergy in terms of both the quantity of bimetallic sites and the electronic state alteration. Similarly, the forward scan curve for isopropanol on the Pt_*x*_Rh_*y*_ alloy is divided into two regions (at 0.1 V_RHE_ and 0.5 V_RHE_, respectively). These regions correspond to the electrooxidation at the Pt–Rh bimetallic sites and the Pt–Pt sites. Doping Rh also successfully reduced the onset potential for the electrooxidation of isopropanol. In Fig. S4a, Pt_50_Rh_50_ achieves a current density of 0.1 mA cm^−2^ at 0.1 V_RHE_, which is higher than that of Pt, Pt_75_Rh_25_, and Pt_25_Rh_75_. However, the current density of the Pt_*x*_Rh_*y*_ samples is significantly lower than that of the Pt_*x*_Ru_*y*_ samples.

**Fig. 3:**
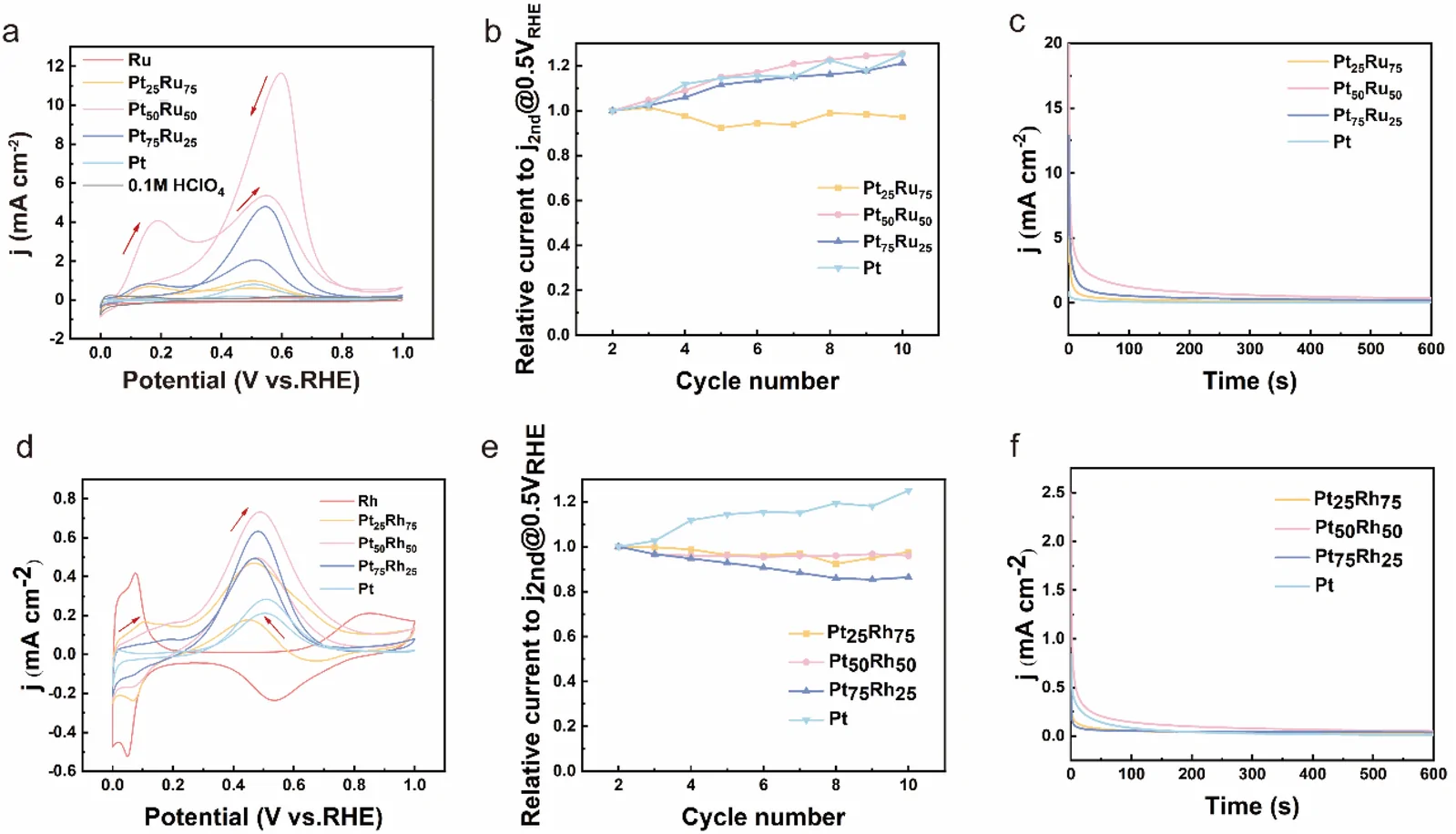
(a) CV curves of Pt, Ru, and Pt_*x*_Ru_*y*_ in 0.1 M HClO_4_ with 1 M isopropanol. (b) Stability test of Pt_*x*_Ru_*y*_ relative to the second-loop current at 0.5 V_RHE_. (c) The chronoamperometry curve at a constant voltage of 0.1 V_RHE_ on Pt_*x*_Ru_*y*_. (d) CV curves of Pt, Rh, and Pt_*x*_Rh_*y*_ in 0.1 M HClO_4_ with 1 M isopropanol. (e) Stability curve of Pt_*x*_Rh_*y*_ relative to the second-loop current at 0.5 V_RHE_. (f) The chronoamperometry curve at a constant voltage of 0.5 V_RHE_ on Pt_*x*_Rh_*y*_.

Additionally, Pt_50_Ru_50_ exhibits a Tafel slope of 52.6 mV dec^−1^, markedly lower than those of Pt (144.05 mV dec^−1^), Pt_75_Ru_25_ (106.78 mV dec^−1^), and Pt_25_Ru_75_ (75.12 mV dec^−1^) (Fig. S3b). The smaller Tafel slope indicates that Pt_50_Ru_50_ exhibits superior electronic conductivity, thereby reducing resistance to electron transfer to active sites and conferring relatively high catalytic activity. The result is consistent with the electrochemical impedance spectroscopy (EIS) data in Fig. S3c, where the charge-transfer resistances for Pt, Pt_75_Ru_25_, Pt_50_Ru_50_, Pt_25_Ru_75_, and Ru are 803.4 Ω, 409.7 Ω, 182.2 Ω, 204.5 Ω, and 189.6 Ω, respectively. Thus, Pt_50_Ru_50_ exhibits the lowest charge-transfer resistance, resulting in a faster charge-transfer rate. The same trend is observed in Pt_*x*_Rh_*y*_ samples. The Tafel slope of Pt_50_Rh_50_ is 99.29 mV dec^−1^, which is significantly lower than that of Pt (135.48 mV dec^−1^), Pt_75_Rh_25_ (172.63 mV dec^−1^), and Pt_25_Rh_75_ (111.48 mV dec^−1^) (Fig. S4b). Pt_50_Rh_50_ exhibits a smaller semicircle than Pt, Pt_75_Rh_25_, Pt_25_Rh_75_, and Rh in the Nyquist plot. The charge transfer resistances for Pt, Pt_75_Rh_25_, Pt_50_Rh_50_, Pt_25_Rh_75_, and Rh are 803.4 Ω, 652.3 Ω, 69.4 Ω, 698.7 Ω, and 101.2 Ω, respectively (Fig. S4c). In summary, Pt_50_M_50_ (M = Ru, Rh) exhibits the lowest onset potential and optimal electrocatalytic performance due to achieving an optimal balance between the number of Pt–M bimetallic sites and electronic conductivity.

The stability of the Pt_*x*_M_*y*_ alloy was assessed by two methods, one of which involved comparing the variation trends of each sample relative to the second-cycle current density at 0.5 V_RHE_ under identical scan rates (Fig. 3b and e).^32^ The CV curve diagram for multiple cycles is shown in Fig. S5. The current densities of Pt, Pt_75_Ru_25_, and Pt_50_Ru_50_ increase slightly with scan cycles, likely due to surface reconstruction beneficial for isopropanol oxidation. The growth trend for Pt_25_Ru_75_ is less pronounced than that of the other samples, while Pt_50_Ru_50_ exhibits the most significant increase. On the other hand, the current densities of Pt_25_Rh_75_, Pt_50_Rh_50_, and Pt_75_Rh_25_ decrease with increasing scan cycle (Fig. 3e), without any promoting surface reconstruction during CV cycles. The second stability test was *via* the long-term chronoamperometry method (Fig. 3c). Pt_50_Ru_50_ exhibits the least current density loss after 600 seconds, outperforming other catalysts. In contrast, Rh-doped samples showed a rapid decline in activity, losing 96% of their current density at 50 seconds. Overall, Ru doping not only promotes surface restructuring and increases current density during CV activation but also exhibits superior resistance to degradation under long-term constant-potential testing.

The differences in electrocatalytic performance are deeply rooted in the electronic and surface properties of the Pt_*x*_Ru_*y*_ and Pt_*x*_Rh_*y*_ catalysts. The surface electronic properties of Pt_*x*_M_*y*_ were investigated by X-ray photoelectron spectroscopy (XPS). As shown in Fig. 4a and b, the Ru 3p spectrum of Pt_50_Ru_50_ is deconvoluted into four peaks: metallic Ru^0^ at 462.0 eV (3p_3/2_) and 484.0 eV (3p_1/2_) alongside oxidized Ru^4+^ at 464.4 eV (3p_3/2_) and 489.8 eV (3p_1/2_). Compared to the standard binding energies for Ru^0^ (462.3 eV for 3p_3/2_ and 485.0 eV for 3p_1/2_), the observed Ru^0^ peaks are shifted to lower values. The Pt 4f spectrum of Pt_50_Ru_50_ is deconvoluted into four peaks: metallic Pt^0^ at 71.5 eV (4f_7/2_) and 74.8 eV (4f_5/2_), and oxidized Pt^2+^ at 73.5 eV (4f_7/2_) and 76.3 eV (4f_5/2_). The Pt^0^ 4f_7/2_ peaks for all three Pt_*x*_Ru_*y*_ are shifted to higher binding energies relative to the standard value for Pt^0^ (71.2 eV). These observations indicate electron transfer from Pt to Ru in the bimetallic alloy, consistent with the electronegativity differences between the two elements (Pt: 2.28; Ru: 2.20), resulting in the formation of electron-deficient Pt species and electron-rich Ru species on the catalyst surface. As the ruthenium doping concentration increases, platinum atoms are progressively replaced by ruthenium in their neighboring regions. This enhances the platinum–ruthenium interactions and promotes electron transfer from platinum to ruthenium. XPS reveals a persistent positive shift in the platinum 4f bonding energy and a persistent negative shift in the ruthenium 3p bonding energy (Table S5), indicating a continuous enhancement of electron transfer during ruthenium doping.

**Fig. 4:**
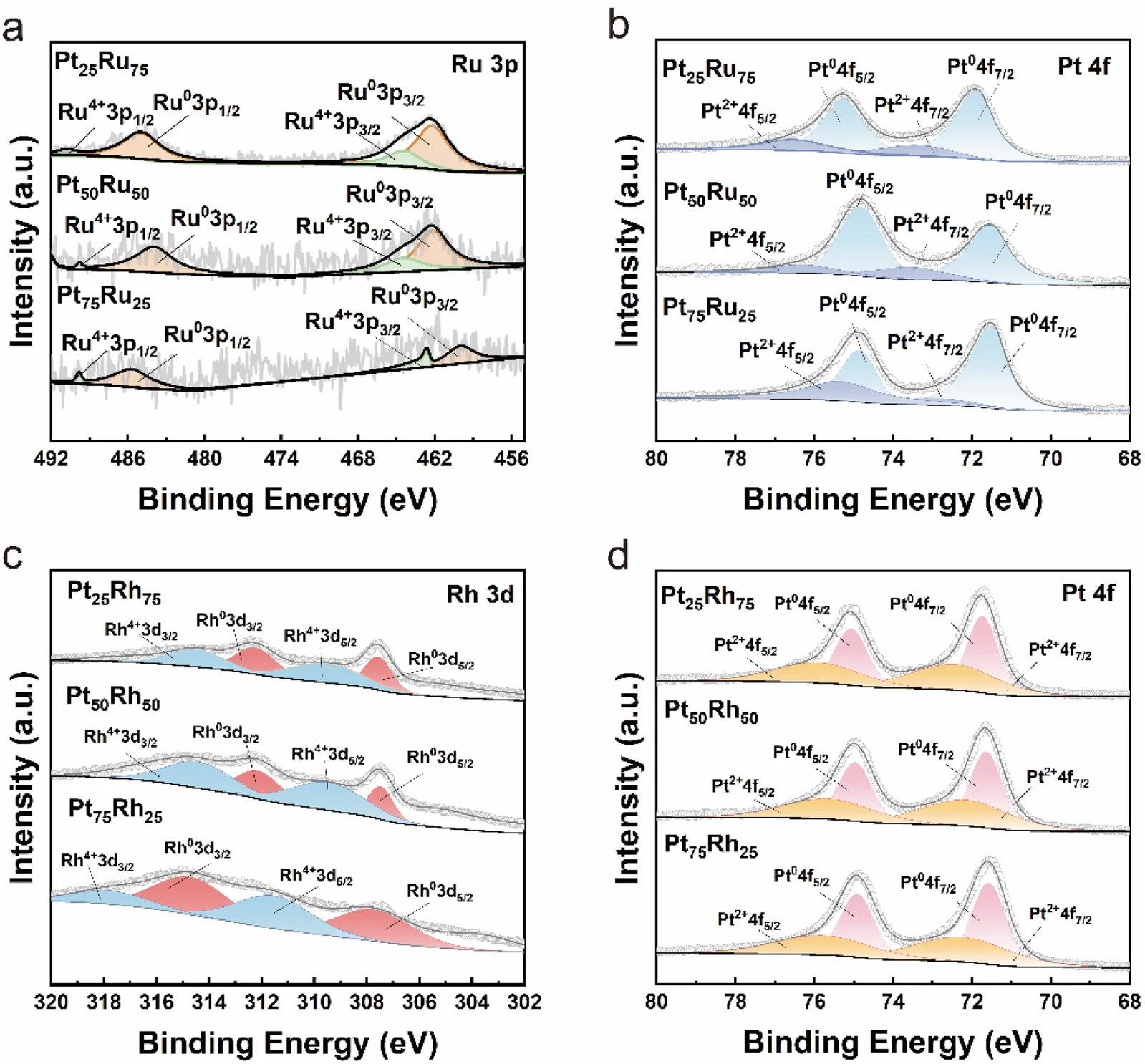
The deconvoluted XPS spectra of Pt_*x*_M_*y*_: (a) and (b) Pt_*x*_Ru_*y*_, (c) and (d) Pt_*x*_Rh_*y*_.

The same phenomenon is observed on the Pt_*x*_Rh_*y*_ catalyst, as shown in Fig. 4c and d. the Rh 3d spectrum of Pt_50_Rh_50_ is deconvoluted into four peaks: metallic Rh^0^ at 307.5 eV (3d_5/2_) and 312.2 eV (3d_3/2_) alongside oxidized Rh^4+^ at 309.6 eV (3d_5/2_) and 314.4 eV (3d_3/2_). Compared to the standard binding energies for Rh^0^ (307.6 eV for 3d_5/2_ and 312.3 eV for 3d_3/2_), the observed Rh^0^ peaks are shifted to lower values. Compared to the standard binding energies for Pt^0^ (71.2 eV for 4f_7/2_ and 74.5 eV for 4f_5/2_), the observed Pt^0^ peaks are shifted to higher values (Table S6). The transfer of electrons from platinum (Pt) to rhodium (Rh) leads to the formation of electron-deficient platinum species and electron-rich rhodium species on the catalyst surface.

The continuous positive shift in the Pt 4f binding energy and the continuous negative shift in the M (M = Ru and Rh) 3p binding energy in XPS indicate an increase in the degree of electron transfer from Pt to M. This electronic structural evolution renders the Pt site electron-deficient, favoring weakened binding to strongly adsorbed acetone, thereby preventing the active sites from becoming over-occupied.^36,37^ Conversely, the electron-rich M site promotes the formation of surface hydroxyl or oxide species at lower potentials, providing auxiliary reaction sites that promote the transformation and also the oxidative desorption of poisoning acetone.^38^ It is worth noting that the Pt_50_M_50_ (M = Ru and Rh) sample exhibits a relatively balanced electronic structure and a high density of bimetallic sites, synergistically optimizing both reaction kinetics and oxidative acetone desorption. The electron-deficient Pt sites and electron-rich M sites are in close proximity, jointly lowering the reaction initiation potential and accelerating electron transfer during key reaction steps.^39,40^

Although electron transfer occurs between Pt–Ru and Pt–Rh, there is a certain difference in the oxygen affinity of the doping metal. It has been reported by DFT calculations that the adsorption strength of the OH* intermediate on transition-metal surfaces follows the order Mo > Ru > Rh > Pt > Au.^41^ Ru's higher oxygen affinity makes it easier to form abundant surface oxides and hydroxyl groups and provides additional sites for the oxidation of isopropanol. Rh, on the other hand, has relatively weaker oxygen affinity and therefore struggles to exert a similar promoting effect under equivalent conditions.

Compared to other reported Pt-based electrooxidation catalysts of isopropanol (Table 1). The onset potential of Pt_50_Ru_50_ was reduced to 0.1 V_RHE_, with the maximum peak potential occurring at 0.6 V_RHE_, outperforming most previous studies. The maximum current density achieved in this study was 11 mA cm^−2^, which is relatively low compared with that reported in other studies due to the use of a GC electrode.^42^ Notably, the current density reported in this study is normalized by geometric area rather than electrochemical active surface area (ECSA).

**Table 1:** Comparison of Pt-based catalysts for electro-oxidation of isopropanol

Catalyst	Onset potential/V_RHE_	Peak potential/V_RHE_	Maximum current density/mA cm^−2^
Pt–Ir^43^	0.31	0.75	3.90
Pt–Cu/C^44^	0.60	0.85	20
Pt^29^	0.30	0.70	35
Pt–Pb/C^45^	0.30	0.60	—
Pt–Ni^46^	0.20	0.72	0.62
Pt–Ru/C^30^	0.10	0.75	60
Pt–Ru/C^47^	0.30	0.85	4.10
Pt–Ru–Ir^48^	0.15	0.60	0.40
Pt–Ni/C^24^	0.50	1.70	100
**Pt**_**50**_ **Rh**_**50**_ **(this work)**	**0.05**	**0.50**	**0.7**
**Pt**_**50**_ **Ru**_**50**_ **(this work)**	**0.10**	**0.60**	**11**

### 3.3. Kinetic and thermodynamic mechanisms of isopropanol electrooxidation on Pt_*x*_M_*y*_

To elucidate the kinetic mechanism of isopropanol oxidation on the Pt_*x*_M_*y*_ alloy, CV curves were recorded at scan rates of 10, 50, 100, 150, 200, 250, and 300 mV s^−1^ over the potential range of 0 V_RHE_ to 1 V_RHE_. The ratio of forward to reverse peak currents was compared to analyze the effect of scan rates on isopropanol electrocatalysis. The current density peaks (I) and (II) exhibit linear relationships with the square root of the scan rate. With increasing scan rate, the current ratio of peaks (I) and (II) increases linearly, indicating irreversible charge transfer at the electrode surface during the electrooxidation of isopropanol, and that the reaction is diffusion-controlled. Furthermore, the CV curves are superimposed at the onset of the potential scan, suggesting that the interfacial charge transfer is kinetically controlled (Fig. 5a, d and S6).

**Fig. 5:**
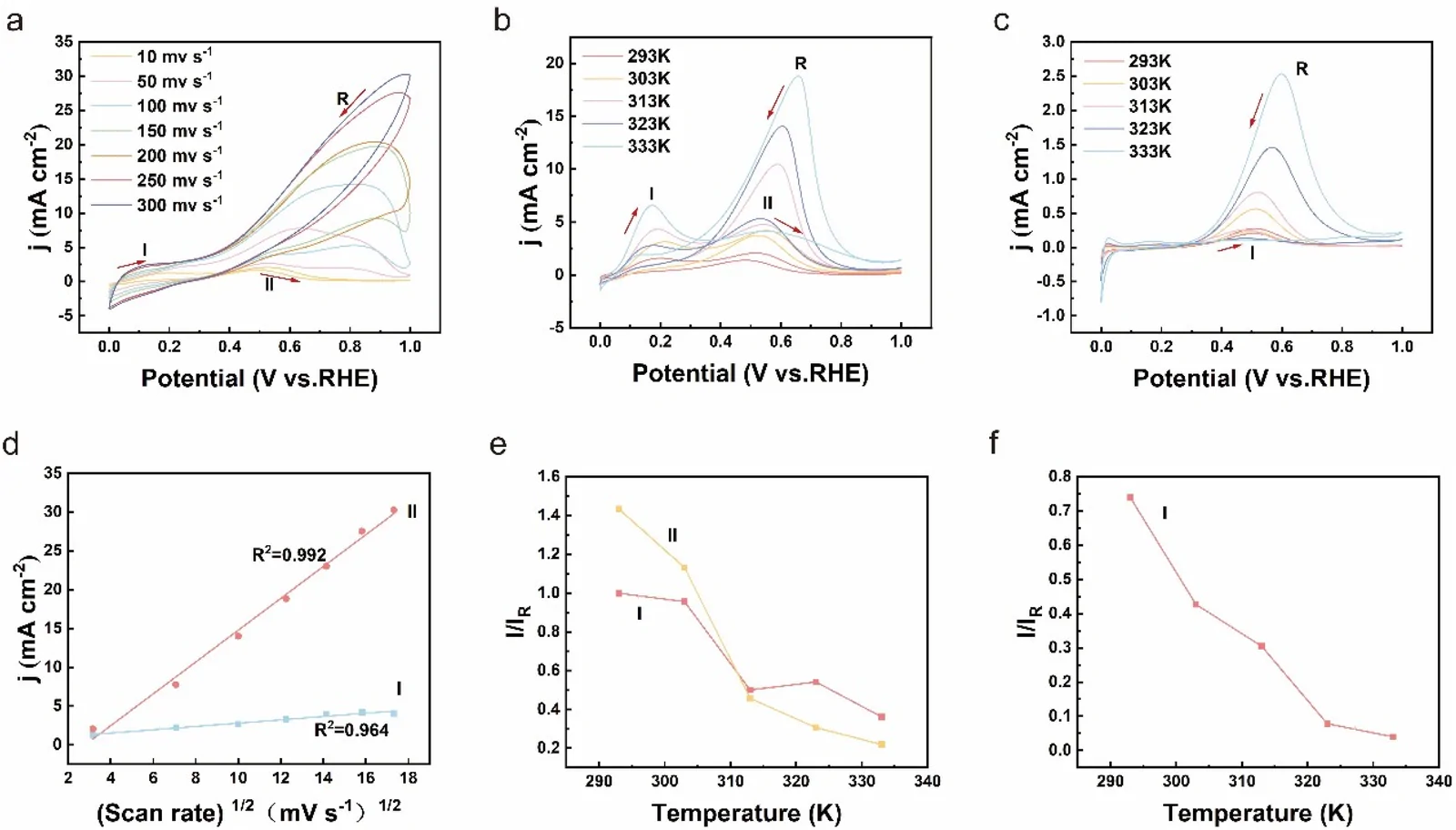
(a) Cyclic voltammetry of Pt_50_Ru_50_ at different scan rates. (b) Cyclic voltammetry curves of Pt_50_Ru_50_ at 293 K, 303 K, 313 K, 323 K, and 333 K. (c) Cyclic voltammetry curves of Pt at 293 K, 303 K, 313 K, 323 K, and 333 K. (d) Linear relationship plot of forward scan peak current *versus* reverse scan peak current for Pt_50_Ru_50_ at different scan rates. (e) Ratio of forward-to-reverse peak currents for Pt_50_Ru_50_. (f) Ratio of forward-to-reverse-scan peak current for Pt.

The thermodynamic mechanism of isopropanol electrooxidation was investigated by conducting CV scans on Pt and Pt_50_M_50_ (M = Ru, Rh) from 293 K to 333 K in 10 K intervals. As the temperature rises, the catalyst's electrode activity for the oxidation of isopropanol increases markedly (Fig. 5b, c and S7). The current density growth trends for Pt and Pt_50_Ru_50_ peaks are almost identical. The ratio of peak current density during forward scanning to that during reverse scanning for both Pt and Pt_50_Ru_50_ decreases (Fig. 5e and f). Because the desorption of the poisoning species, acetone, intensifies with increasing temperature, enhancing the ability of negative potentials to regenerate the poisoned catalyst's surface, Ru doping accelerates this process. Moreover, the peak current density of Pt_50_Ru_50_ during forward scanning is reduced more than during reverse scanning, indicating that the catalytic activity of Pt–Ru active sites diminishes at elevated temperatures. This may be attributed to the partial dissolution of Ru at higher temperatures. As shown in Fig. S5, both log *j* and 1/*T* exhibit linear relationships, indicating that the reaction mechanism at 0.5 V_RHE_ remains unchanged and is unaffected by temperature variations. The activation energies for Pt and Pt_50_Ru_50_ at 0.2 V_RHE_ are then calculated. The activation energy for Pt is 17.55 kJ mol^−1^, while that for Pt_50_Ru_50_ is 12.56 kJ mol^−1^, respectively, indicating that Ru doping thermodynamically promotes the electrocatalytic reaction of isopropanol. Similarly, as shown in Fig. S9, the Pt_*x*_Rh_*y*_ reaction is also diffusion-controlled. As the temperature rises, the electrode activity of the Pt–Pt sites in the Pt_*x*_Rh_*y*_ catalyst for the oxidation of isopropanol increases significantly, but the increase in temperature has a limited effect on enhancing the catalytic activity of the Pt–Rh sites (Fig. S8 and S9).

## 4. Conclusion

In summary, a series of Pt_*x*_M_*y*_ (*x* + *y* = 100, M = Ru or Rh) bimetallic catalysts was successfully synthesized *via* constant-potential electrodeposition. The effects of doping metal and doping amount on the catalyst's phase structure, morphology, electrocatalytic performance, and the mechanism of isopropanol electrooxidation under acidic conditions were investigated. Both Rh and Ru are effective at enhancing the performance of Pt-based catalysts through a bimetallic synergy effect; however, Ru doping has a greater optimizing effect due to its stronger oxygen affinity. Adsorption of oxygen species at ruthenium sites provides auxiliary sites for isopropanol oxidation and oxidative desorption of acetone, thereby enhancing the efficiency and stability of isopropanol oxidation electrocatalysis. Moreover, electrochemical performance tests suggest a correlation between catalytic activity and Ru content: insufficient Ru content leads to inadequate formation of Pt–Ru bimetallic sites, while excess Ru induces alloying defects and weakens intermetallic interactions, thereby limiting the enhanced desorption of acetone intermediates. The Pt_50_Ru_50_ ratio is identified as the optimal composition. This work emphasizes the importance of both bimetallic synergy and oxygen affinity in the electro-oxidation of isopropanol and provides insights into the design of efficient catalysts for electro-active LOHCs. Future work will focus on extending M to other oxygen-affinity elements to investigate whether the trends observed in this paper are equally applicable.

## Conflicts of interest

There are no conflicts to declare.

## Supplementary Material

RA-016-D6RA06258J-s001

## Data Availability

The data supporting this article have been included as part of the supplementary information (SI). Supplementary information: the tables for calculated lattice constants, ICP-AES results, element contents under electromicroscopy, XPS peak values, and XPS fitting parameters of all catalysts; SEM of electrodeposited monometallic Pt, Ru, and Rh; particle size distribution of Pt_*x*_Ru_*y*_ samples; LSV, Tafel plots, and EIS of Pt_*x*_Ru_*y*_ and Pt_*x*_Rh_*y*_; multiple-cycle CV diagrams of Pt_*x*_Ru_*y*_ samples; CV curves of Pt_*x*_Ru_*y*_ and Pt_*x*_Rh_*y*_ at different scan rates; CV of Pt_50_Ru_50_ and Pt_50_Rh_50_ under different temperatures and corresponding Arrhenius plots. See DOI: https://doi.org/10.1039/d6ra06258j.
