# Supplementary material for: Ru-specific promotion in electrodeposited Pt-based bimetallic catalysts for isopropanol electrooxidation
Source: RSC Adv. 2026 Sep 7;16(43):53274–83. doi: 10.1039/d6ra06258j (PMC13549307; doi:10.1039/d6ra06258j)
Supplement: Supplementary file 1 [file RA-016-D6RA06258J-s001.pdf]

Supplementary Information (SI) for RSC Advances.
This journal is © The Royal Society of Chemistry 2026

Ru-specific Promotion in Electrodeposited Pt-based Bimetallic Catalysts for Isopropanol Electrooxidation

Supporting Information

Table S1 Calculated lattice constant values for Pt, $Pt_{75}Ru_{25}$, and $Pt_{50}Ru_{50}$.

	Pt	$Pt_{75}Ru_{25}$	$Pt_{50}Ru_{50}$
Lattice constant (a=b=c)/ Å	3.91	3.88	3.86
Unit cell volume/ A^3	60.05	58.57	57.96

Table S2 Calculated lattice constant values for Pt, $Pt_{75}Ru_{25}$, and $Pt_{50}Ru_{50}$.

	Pt	$Pt_{75}Rh_{25}$	$Pt_{50}Rh_{50}$
Lattice constant (a=b=c)/ Å	3.91	3.88	3.87
Unit cell volume/ A^3	60.05	58.74	57.66

Table S3 ICP-AES Test Results.

	$Pt_{75}Ru_{25}$	$Pt_{50}Ru_{50}$	$Pt_{25}Ru_{75}$	$Pt_{75}Rh_{25}$	$Pt_{50}Rh_{50}$	$Pt_{25}Rh_{75}$
Pt/M feeding ratio	3:1	1:1	1:3	3:1	1:1	1:3
Pt/M experimental ratio	8:1	4:1	0.1:1	1.5:1	1.4:1	0.7:1

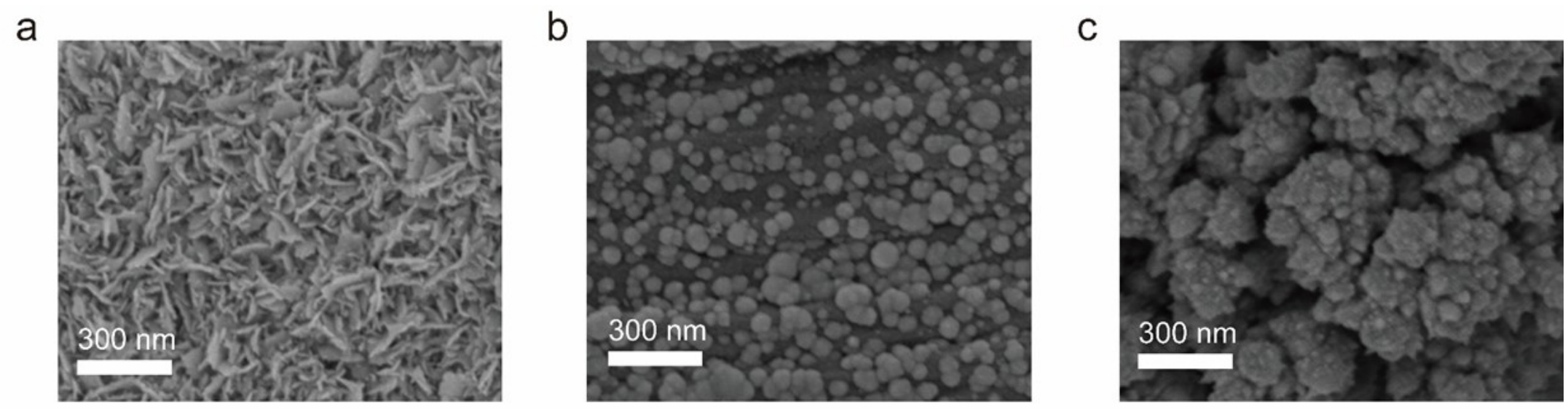

Figure S1 SEM images of different metal samples: (a) Pt, (b) Ru (c) Rh.

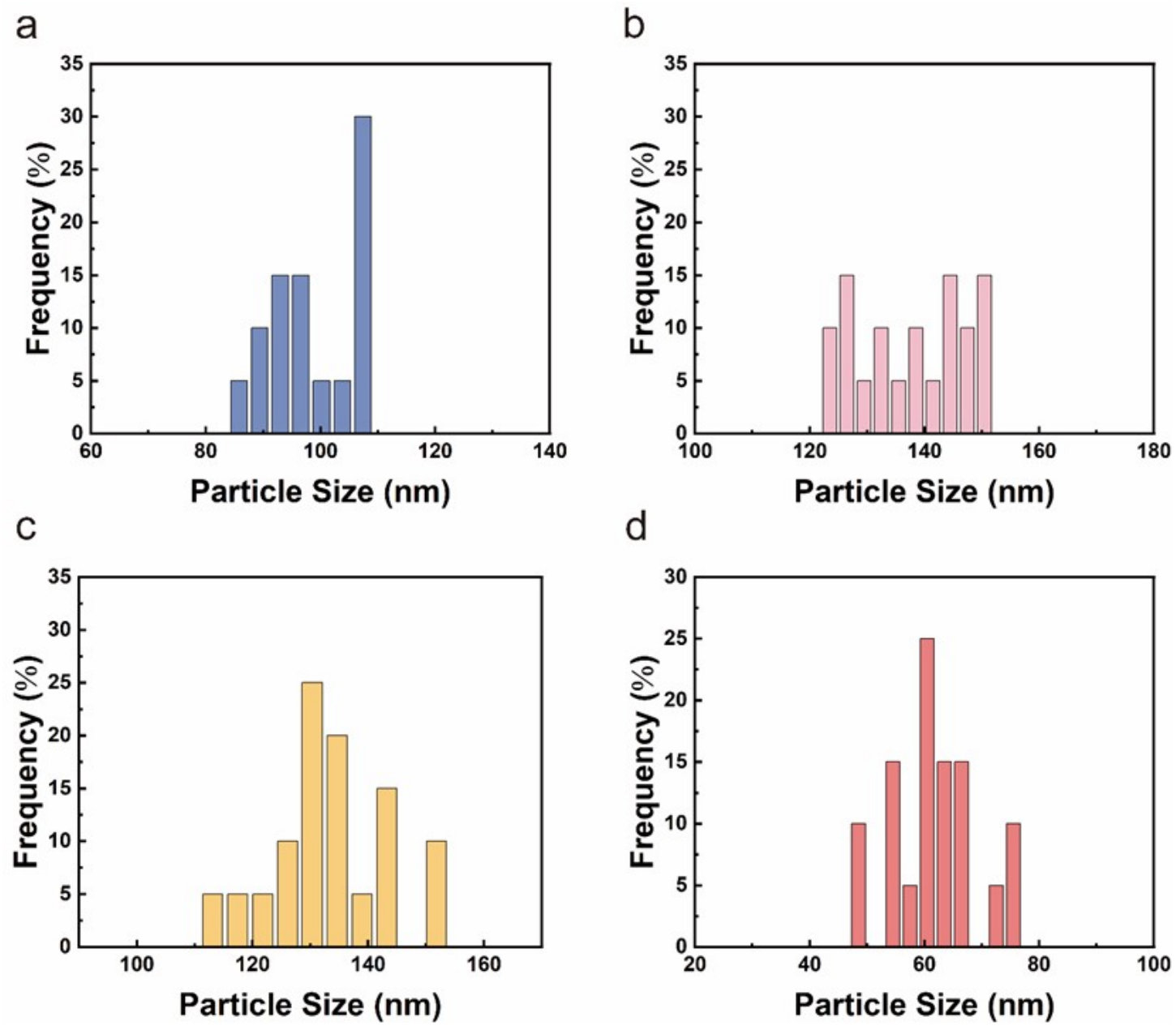

Figure S2 Average particle size distribution of different Pt_xRu_y samples (a) $Pt_{75}Ru_{25}$, (b) Pt50Ru50, (c) Pt25Ru75, (d) Ru.

Table S4 Electron Microscope Element Content.

Element	Line type	K Factor	Absorption Correction	Wt%	Wt% Sigma	Atomic %
Ru	L series	1.820	1.00	27.64	0.38	42.44
Pt	L series	2.266	1.00	72.36	0.38	57.66
Total				100		100

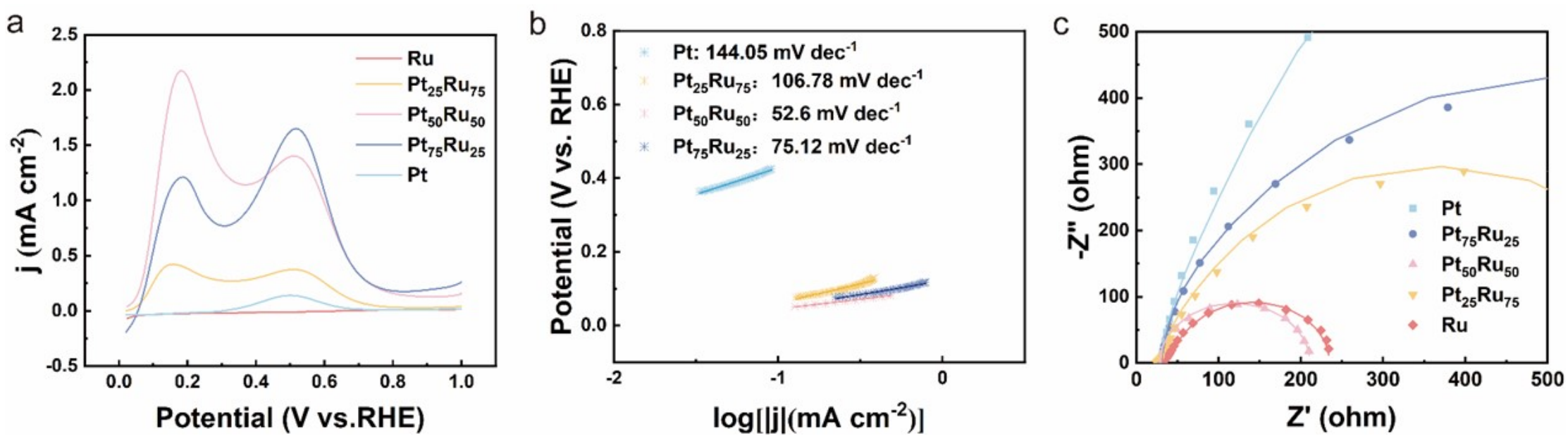

Figure S3 (a) LSV curves of Pt, Ru, and Pt_xRu_y in 0.1 M $HClO_4$ with 1 M isopropanol. (b) Corresponding Tafel slopes of the LSV curves. (c) EIS of Pt_xRu_y at -0.2 V_{RHE}.

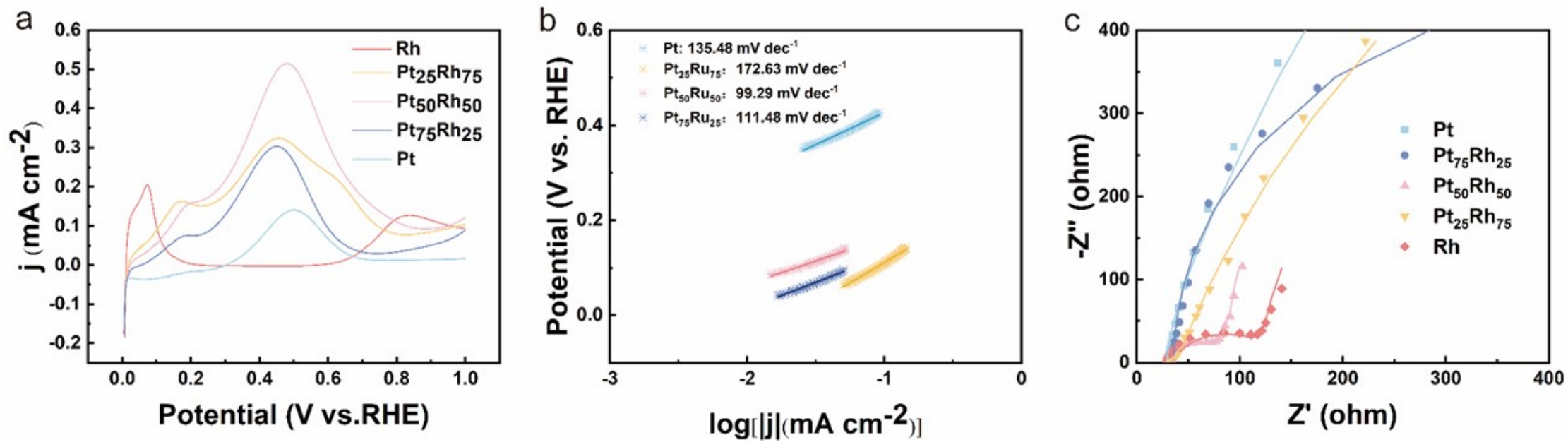

Figure S4 (a) LSV curves of Pt, Rh, and Pt_xRh_y in 0.1 M $HClO_4$ with 1 M isopropanol. (b) Corresponding Tafel slopes of the LSV curves. (c) EIS of Pt_xRu_y at -0.2 V_{RHE}.

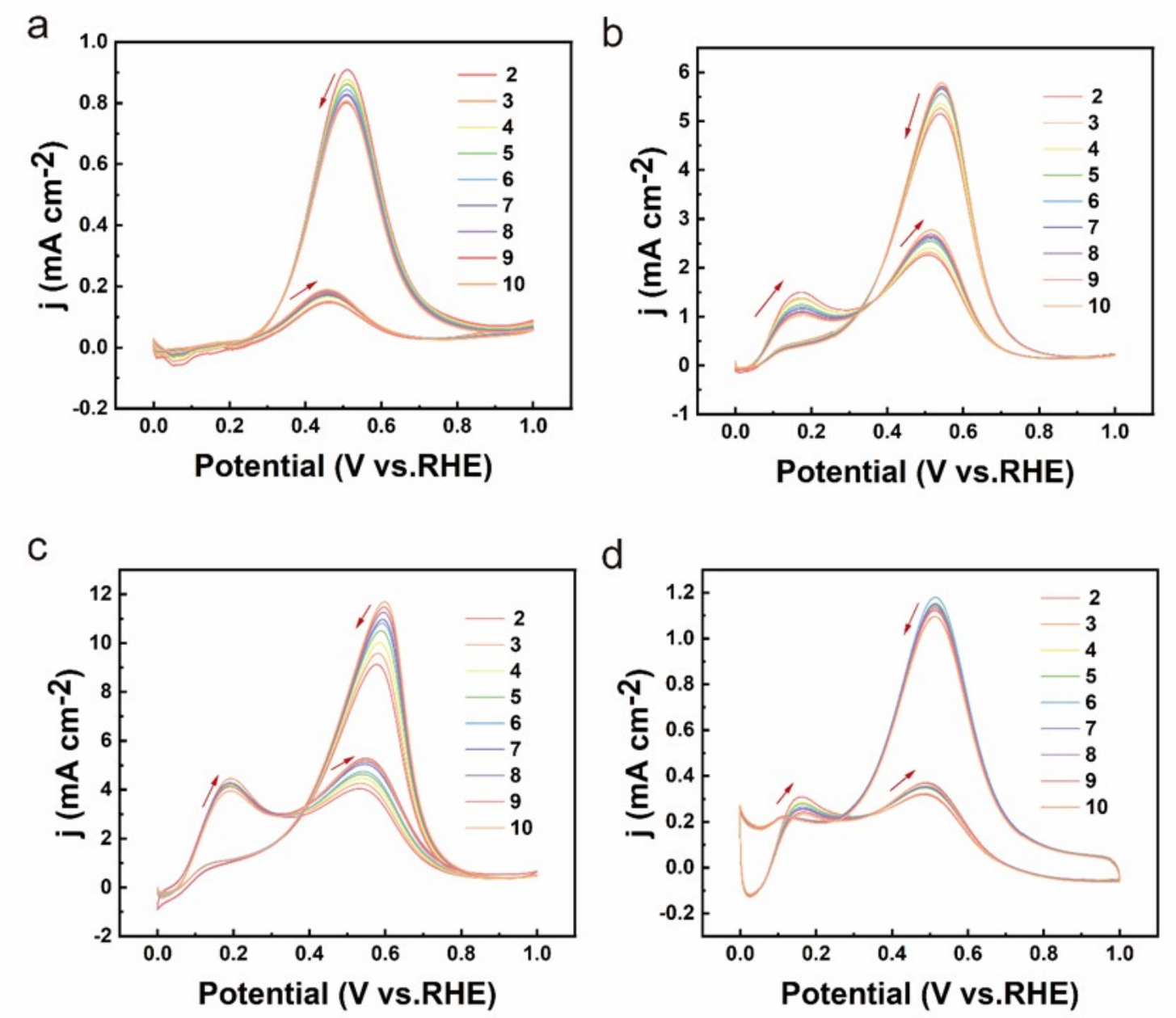

Figure S5 Multiple-cycle CV diagrams of different Pt_xRu_y samples (a) Pt, (b) $Pt_{75}Ru_{25}$, (c) $Pt_{50}Ru_{50}$, (d) $Pt_{25}Ru_{75}$.

Table S5 Ru^0 and Ru^{4+} Peak Values for Different XPS Samples.

Sample	$Ru^0(Ru3p_{3/2})$ /eV	$Ru^0(Ru3p_{1/2})$ /eV	$Ru^{4+}(Ru3p_{3/2})$ /eV	$Ru^{4+}(Ru3p_{1/2})$ /eV
$Pt_{25}Ru_{75}$	462.2	485.0	464.2	490.8
$Pt_{50}Ru_{50}$	462.0	484.0	464.4	489.8

Pt^0 and Pt^{2+} Peak Values for Different XPS Samples.

Sample	$Pt^0(Pt4f_{7/2})$ /eV	$Pt^0(Pt4f_{5/2})$ /eV	$Pt^{2+}(Pt4f_{7/2})$ /eV	$Pt^{2+}(Pt4f_{5/2})$ /eV
$Pt_{25}Ru_{75}$	71.2	75.3	73.4	76.5
$Pt_{50}Ru_{50}$	71.5	74.8	73.5	76.3
$Pt_{75}Ru_{25}$	71.5	74.8	72.8	75.3

Table S6 Rh^0 and Rh^{4+} Peak Values for Different XPS Samples.

Sample	Rh^0($Rh3d_{5/2}$) /eV	Rh^0($Rh3d_{3/2}$) /eV	Rh^{4+}($Rh3d_{5/2}$) /eV	Rh^{4+}($Rh3d_{3/2}$) /eV
$Pt_{25}Rh_{75}$	307.6	312.3	309.7	314.6
$Pt_{50}Rh_{50}$	307.5	312.2	309.5	314.4
$Pt_{75}Rh_{25}$	307.8	314.8	311.4	317.9

Pt^0 and Pt^{2+} Peak Values for Different XPS Samples.

Sample	Pt^0($Pt4f_{7/2}$) /eV	Pt^0($Pt4f_{5/2}$) /eV	Pt^{2+}($Pt4f_{7/2}$) /eV	Pt^{2+}($Pt4f_{5/2}$) /eV
$Pt_{25}Rh_{75}$	71.8	72.5	75.1	76.0
$Pt_{50}Rh_{50}$	71.7	72.3	75.0	75.8
$Pt_{75}Rh_{25}$	71.6	72.4	74.9	75.9

Table S7 XPS fitting parameters for Pt-Ru catalysts.

Parameter	$Pt_{25}Ru_{75}$	$Pt_{50}Ru_{50}$	$Pt_{75}Ru_{25}$
Pt^o $4f_{7/2}$ (FWHM/eV)	1.19	1.3	1.1
Pt^o $4f_{5/2}$ (FWHM/eV)	1.23	1.26	1.06
Area ratio ($4f_{7/2}$:$4f_{5/2}$)	1.30:1	0.98:1	1.45:1
Ru^o $3p_{3/2}$ (FWHM/eV)	3.27	0.96	-
Ru^o $3p_{1/2}$ (FWHM/eV)	3.48	0.83	-
Area ratio ($3p_{3/2}$:$3p_{1/2}$)	1.74:1	1.81:1	-
Pt (PP At.%)	0.48	0.44	0.40
Ru (PP At.%)	0.53	0.36	-
Surface Pt:Ru ratio	0.90:1	1.22:1	-

Table S8 XPS fitting parameters for Pt-Rh catalysts.

Parameter	$Pt_{25}Rh_{75}$	$Pt_{50}Rh_{50}$	$Pt_{75}Rh_{25}$
Pt^o $4f_{7/2}$ (FWHM/eV)	1.09	1.18	1.13
Pt^o $4f_{5/2}$ (FWHM/eV)	1.19	1.23	1.15
Area ratio ($4f_{7/2}$:$4f_{5/2}$)	1.19:1	1.24:1	1.18:1
Rh^o $3d_{5/2}$ (FWHM/eV)	1.00	0.86	0.68
Rh^o $3d_{3/2}$ (FWHM/eV)	1.06	1.02	0.71
Area ratio ($3d_{5/2}$:$3d_{3/2}$)	1.60:1	1.61:1	1.50:1
Pt (PP At.%)	0.95	1.17	1.60
Rh (PP At.%)	2.78	1.18	0.98
Surface Pt:Rh ratio	0.34:1	0.99:1	1.63:1

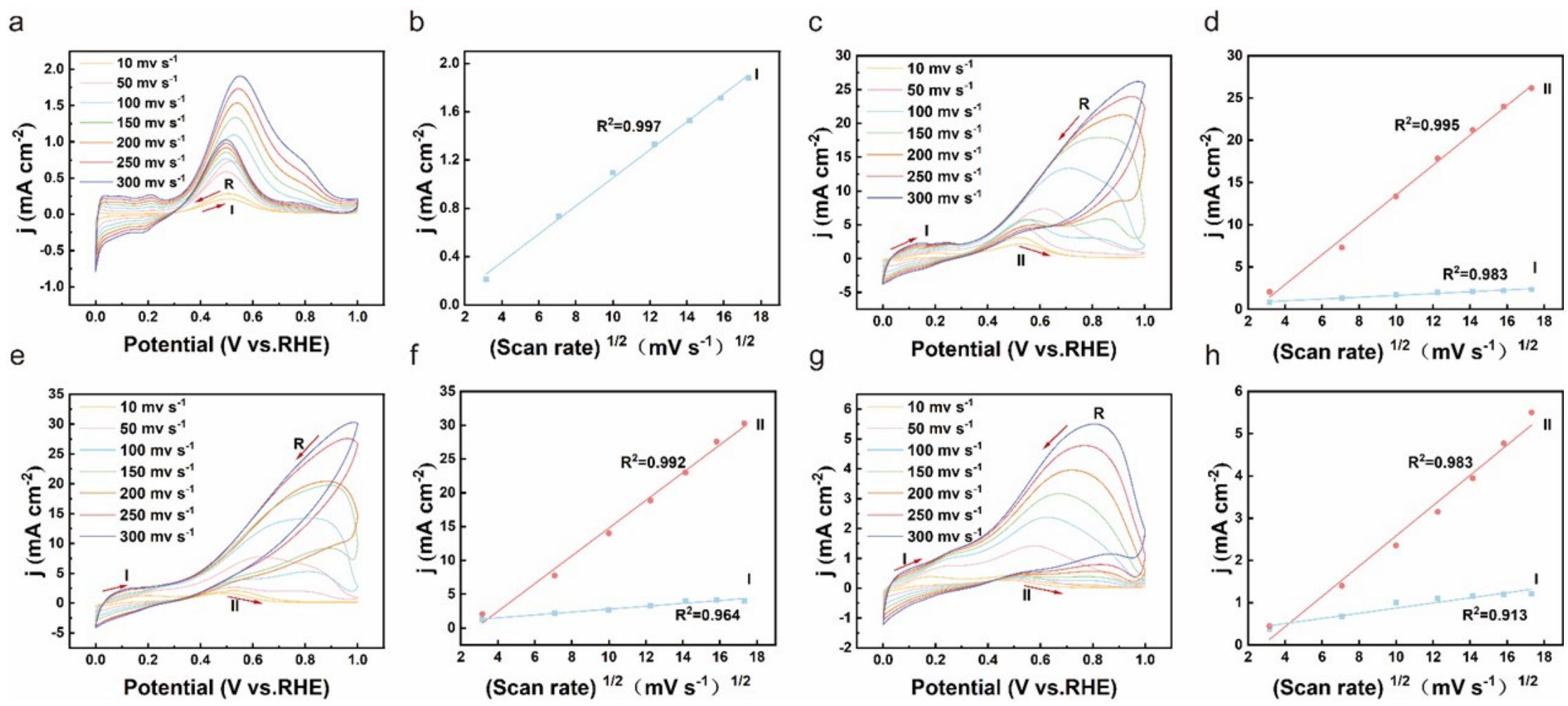

Figure S6 CV curves of Pt_xRu_y at different scan rates and linear relationship plots between forward scan peak current and reverse scan peak current: Pt (a-b), $Pt_{75}Ru_{25}$ (c-d), $Pt_{50}Ru_{50}$ (e-f), $Pt_{25}Ru_{75}$ (g-h).

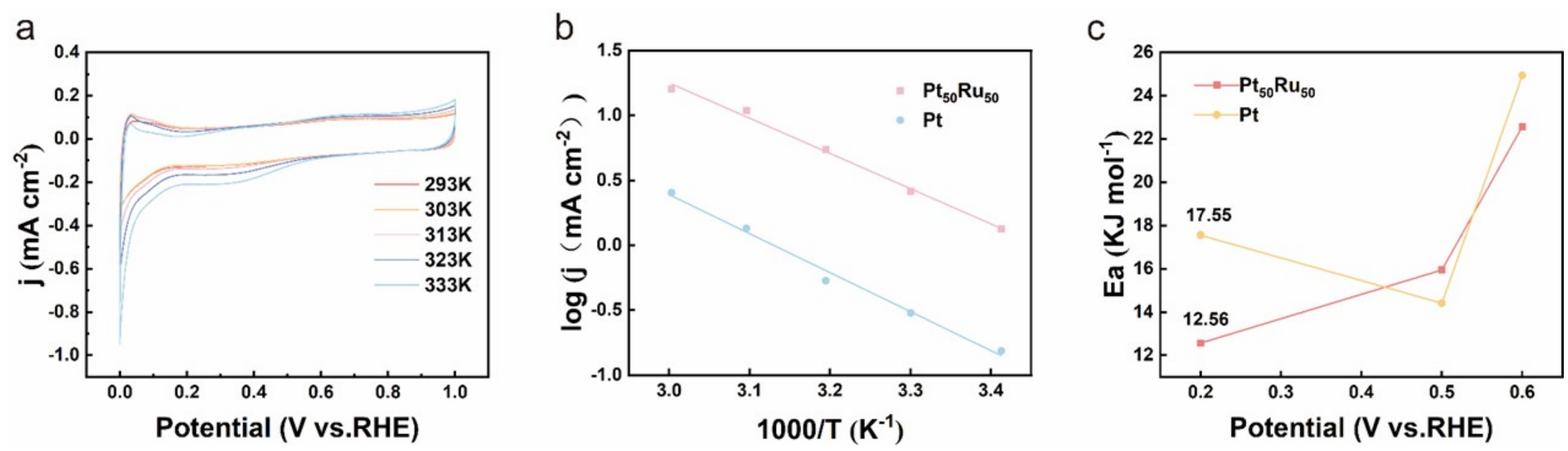

Figure S7 (a) Cyclic voltammetry curves of Ru at 293 K, 303 K, 313 K, 323 K, and 333 K (b) Arrhenius plots for Pt and $Pt_{50}Ru_{50}$ at different temperatures for 0.5 V_{RHE}, (c) Pt and $Pt_{50}Ru_{50}$ activation energy at 0.2 V_{RHE}.

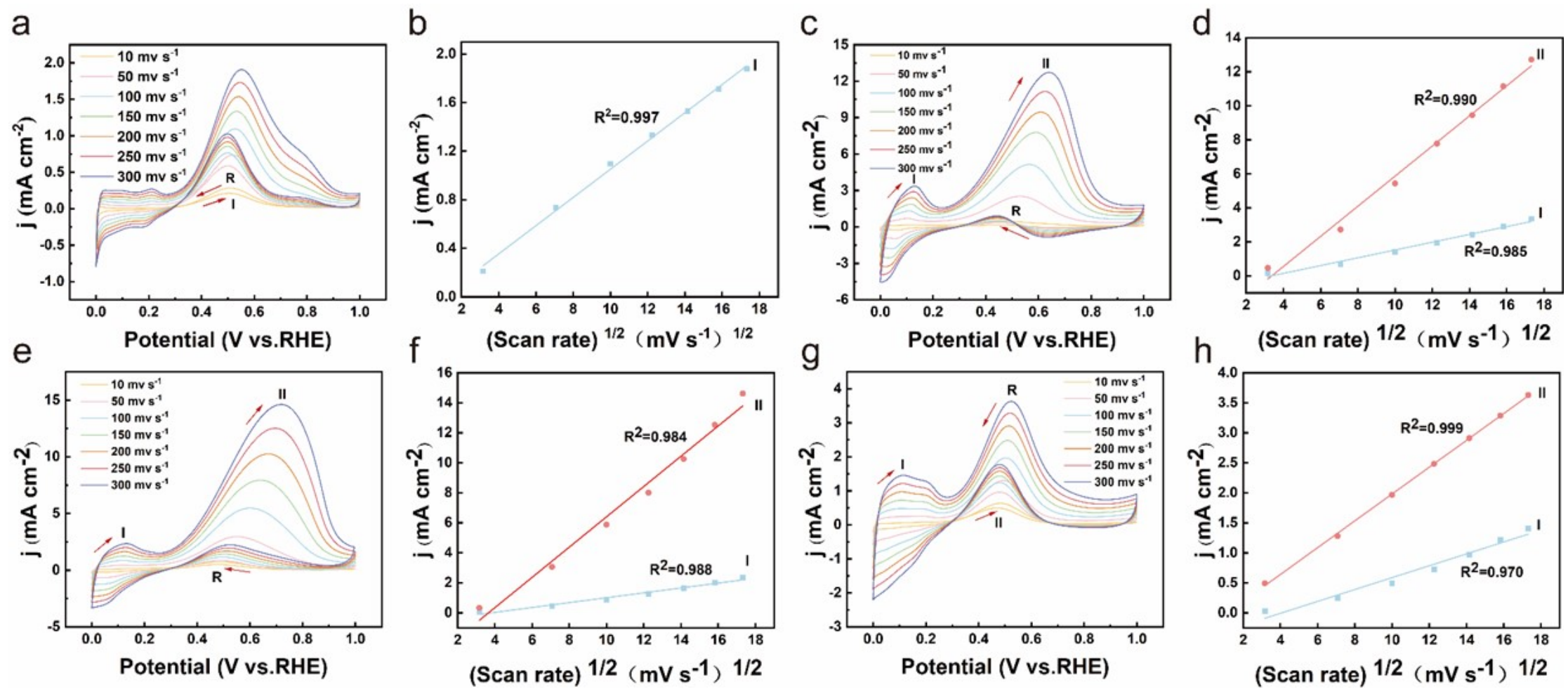

Figure S8 CV curves of Pt_xRh_y at different scan rates and linear relationship plots between forward scan peak current and reverse scan peak current: Pt (a-b), $Pt_{75}Rh_{25}$ (c-d), $Pt_{50}Rh_{50}$ (e-f), $Pt_{25}Rh_{75}$ (g-h).

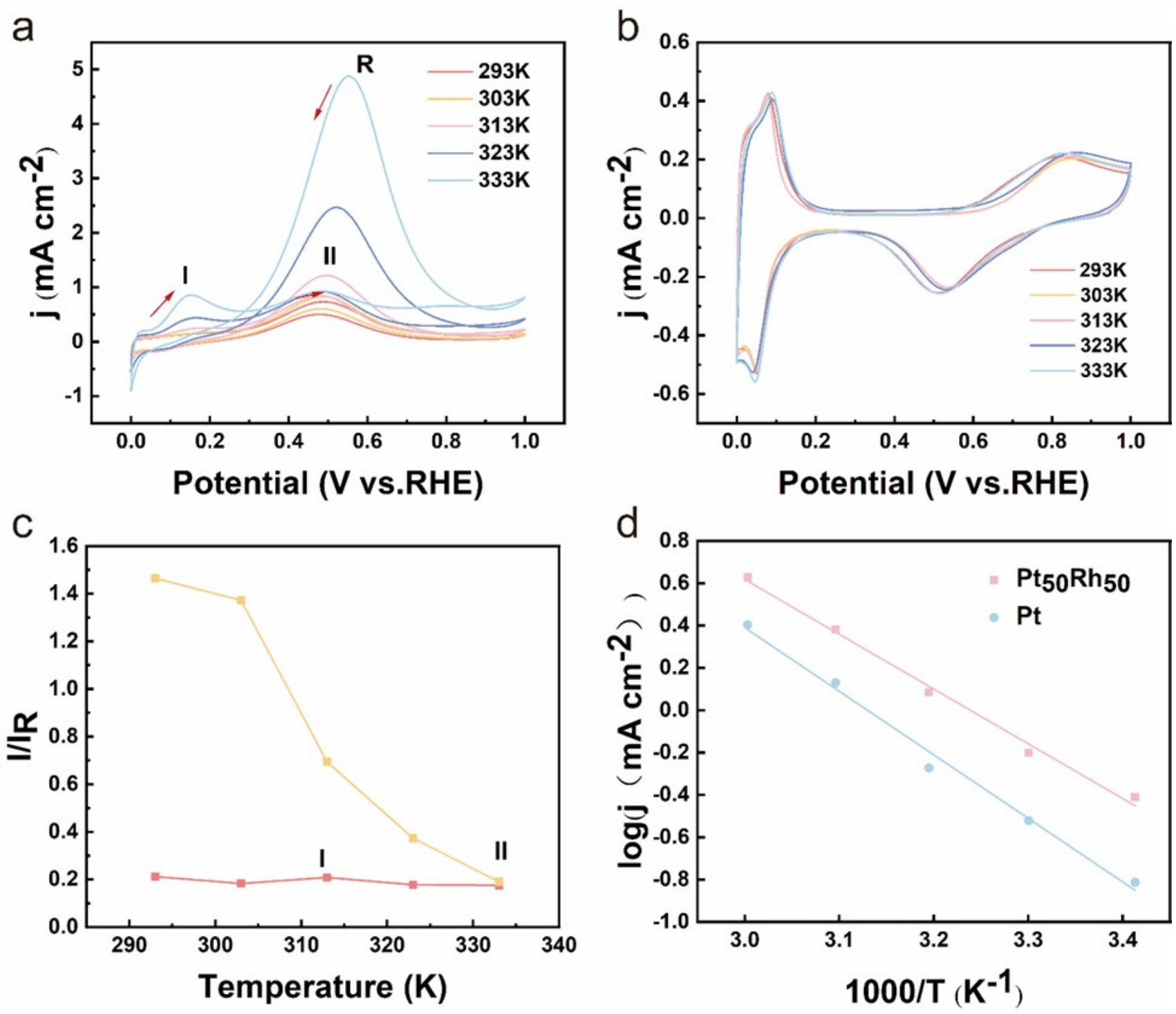

Figure S9 (a) Cyclic voltammetry curves of $Pt_{50}Rh_{50}$ at 293 K, 303 K, 313 K, 323 K, and 333 K. (b) Cyclic voltammetry curves of Rh at 293 K, 303 K, 313 K, 323 K, and 333 K. (c) Ratio of forward to reverse peak current for $Pt_{50}Rh_{50}$. (d) Arrhenius plots for Pt and $Pt_{50}Rh_{50}$ at different temperatures for 0.5 V_{RHE}.
